# Expanding contingency learning models of aggression in youth: Conceptual and methodological considerations to enhance clinical utility^☆^

**DOI:** 10.1016/j.avb.2025.102082

**Published:** 2025-09-09

**Authors:** Amy L. Byrd, Isabella Kahhale, Colin E. Vize, Rebecca Griffith, Essi Viding, Stephanie D. Stepp

**Affiliations:** a University of Pittsburgh School of Medicine, Department of Psychiatry, Pittsburgh, PA, USA; b University of Pittsburgh, Department of Psychology, Pittsburgh, PA, USA; c University College London, Division of Psychology, London, England, United Kingdom of Great Britain and Northern Ireland

**Keywords:** Aggressive behavior, Conditioning, Neural, Physiological, Neurobiological, Ambulatory assessment, Intervention

## Abstract

Aggression in youth is a transdiagnostic indicator that permeates nearly all psychiatric disorders and is one of the most common reasons for mental health referrals. Contingency learning theories provide a framework for conceptualizing factors that elicit (*antecedents*) and maintain aggressive (*consequences*) behavior. For decades, theoretical and etiological models of aggression have emphasized individual differences in neurobiological reactivity during contingency learning as predictors of aggression in youth. However, our ability to predict precisely *when* aggression will occur and *why* aggression persists over time remains limited, ultimately hindering our capacity to tailor and personalize interventions to maximize effectiveness. The current review summarizes research examining individual differences in neurobiological reactivity during stimulus-response (*antecedents*) and response-outcome (*consequences*) contingencies as predictors of aggression in youth. It then offers concrete recommendations for expansions of this work with an eye toward optimizing the prediction of aggression via the identification of proximal individual- and dyad-level antecedents and consequences. These include conceptual considerations such as examining aggression as a transdiagnostic construct, considering alternative internal and external antecedents and consequences of aggression, and explicating aggression within the dyadic context. Additionally, recommendations for methodological advancements are presented, including enhancing ecological validity of study designs, incorporating ambulatory assessments, and utilizing advanced analytic approaches that allow us to empirically test and identify proximal antecedents and consequences of aggression. Finally, we discuss how these advancements have the potential to increase the precision of intervention efforts to reduce aggression by creating a framework for systematically mapping within-individual and within-dyad processes that elicit and maintain aggressive behavior over time.

## 1. Introduction

Aggression refers to a set of behaviors that often emerge within the context of interpersonal conflict and cause harm to others (Anderson & Bushman, 2002; Baron & Richardson, 1994). Aggression in youth is a transdiagnostic indicator that permeates nearly all psychiatric disorders and represents one of the most common reasons for mental health referrals (Kazdin, 2003). The persistence of aggression across development is linked to severe and intractable trajectories of mental illness (Loeber & Hay, 1997; Ostrov & Houston, 2008; Schaeffer et al., 2003; Scott et al., 2014) as well as lower academic achievement, difficulties with interpersonal functioning, and increased risk for suicide, substance use, violence, and incarceration (Brent, 1995; Brook et al., 1992; Conner et al., 2003; Fite et al., 2007; Huesmann et al., 2009; Kokko et al., 2006). Taken together, the annual economic impact of youth aggression is estimated to be over $10 billion in the United States alone (Parker et al., 2024). The high financial cost, prevalence, and associated risks provide strong impetus for improving etiological and intervention models of aggression. However, there has been drastically less research investment in understanding the etiology and treatment of aggression, relative to other common mental health problems. Moreover, our ability to predict precisely *when* aggression will occur and *why* aggression persists over time is still limited. If we want to achieve enhanced precision, we need to reliably identify proximal *antecedents* (what elicits aggression) and *consequences* (what maintains aggression) within daily interpersonal interactions.

Contingency learning theories provide a useful framework for conceptualizing factors that elicit aggressive behavior (*antecedents*) and those that maintain aggressive behavior (*consequences*). Decades of research examining individual differences in neurobiological reactivity to these contingencies have linked physiological and neural reactivity (or lack thereof) to risk for broader externalizing behaviors, including aggression (e.g., Bertsch et al., 2020; Blankenstein et al., 2022; Byrd et al., 2014; Fanning et al., 2017; Fanti et al., 2019; Lorber, 2004). While this work has undoubtedly enhanced our understanding of the potential neurobiological underpinnings of aggression, the focus on externalizing behaviors, a notably heterogeneous construct, is limiting in that aggression is not a *necessary* component (Dodge et al., 2008; Olson et al., 2018). Additionally, the quantification of risk has been focused on distal factors using variable-centered, between-person analyses, which tells us who has higher levels of aggression concurrently and/or who may be more likely to engage in aggression over longer, non-specific periods of time (e.g., 1-year follow-up; Frick & White, 2008; Perkins et al., 2022; Vize et al., 2019). Moreover, research examining neurobiological reactivity to these contingencies has focused primarily on individual-level antecedents and consequences while ignoring potentially potent dyadic factors that may elicit and maintain aggressive behavior over time. Our lack of specificity and limited understanding of within-individual changes in sensitivity to momentary individual- and dyad-level contingencies renders us unable to proximally predict *when* an individual will engage in aggression (antecedents) and *why* they continue to do so (consequences), ultimately hindering our ability to personalize interventions to maximize effectiveness.

The current review first describes contingency learning models as a means for understanding how aggression is elicited and maintained over time. Second, it summarizes empirical work examining individual differences in physiological and neural reactivity during contingency learning as predictors of aggression in youth. Next, we offer specific recommendations for conceptual and methodological expansions of this research with an eye toward optimizing the prediction of aggression via the identification of proximal individual- and dyad-level antecedents and consequences. Finally, we discuss how these expansions have the potential to increase the precision of intervention efforts designed to reduce aggression by creating a framework for systematically mapping within-individual and within-dyad processes that elicit and maintain aggressive behavior over time.

## 2. Contingency learning models of aggression

Contingency learning models, grounded in classical and operant conditioning principles, provide a framework for predicting *when* aggression will be elicited and *why* aggression is maintained (Bouton, 2007; Pavlov, 1927; Rescorla & Solomon, 1967; Skinner, 1969). These models expand on social learning theory which conceptualizes aggression as an imitated behavior acquired from observing one’s social environment (Akers, 1998; Bandura, 1978). While social learning theory offers a means for understanding how aggression can be introduced into one’s behavioral repertoire and how resulting cognitive schemas may increase the likelihood of its recurrence, it provides very little information about the *momentary* contingencies that predict *when* aggression will occur and *why* it persists over time. Contingency learning models emphasize a focus on the assessment of dynamic, momentary changes to identify proximal *antecedents* (stimulus-response; i.e., classical conditioning) and *consequences* (response-outcome; i.e., operant conditioning) within individuals and across interpersonal contexts. These models stem from evolutionary theories that conceptualize aggression from an interactionist perspective whereby aggression arises within the context of a unique set of environmental circumstances and functions as an adaptive solution for survival (Archer, 2009; Buss & Shackelford, 1997). Though aggression in youth is rarely necessary for survival and carries costly and dangerous long-term consequences, these models aim to explain instances of impulsive, emotionally driven forms of aggression (i.e., reactive aggression) and occurrences of more premeditated forms of aggression (i.e., proactive aggression), both of which can be elicited by specific environmental stimuli and function to meet the more immediate needs of an individual (Blair, 2016; Wrangham, 2018). While there continues to be debate about the extent to which contingency learning models fully explain aggressive behavior (Anderson & Bushman, 2002), there is a consensus that more precise characterization of these contingencies has the potential to help us understand what motivates aggressive behavior and enhance prediction of aggression. This, ultimately, can inform more personalized intervention efforts.

**Fig. 1.**
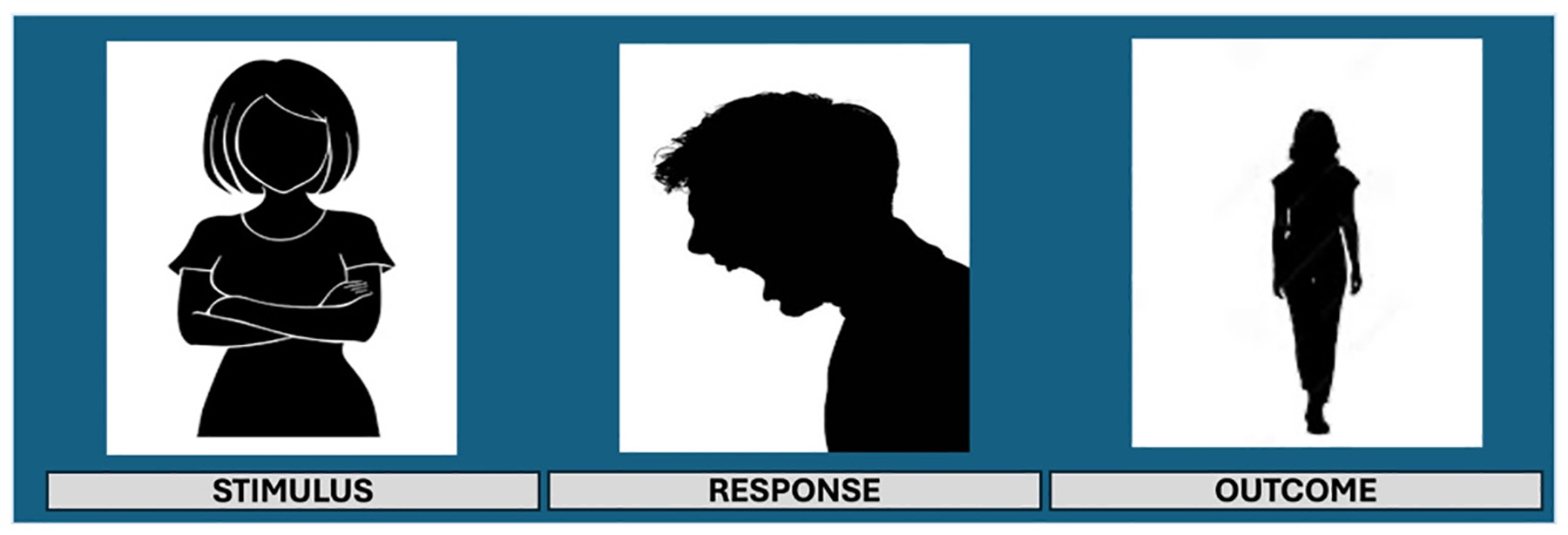
Here we provide an example of a specific **stimulus-response-outcome contingency** that could elicit and reinforce aggression, increasing the likelihood of its reoccurrence over time. **SITUATION**: A 12-year-old who has been struggling socially at school comes home and tells their mom that some kids at school invited them to go to a concert downtown on Saturday night. **STIMULUS**: Mom -with a glare, folded arms, and a stern tone - says “NO! You are not going to a concert by yourself downtown!” This 12-year-old is learning which cues (i.e., conditioned stimulus: glare, folded arms, and a stern tone) predict their mother’s “NO!” response (i.e., *unconditioned stimulus*), which is a threat to desired social engagement and blocking their goal of seeing the concert. **RESPONSE**: The 12-year-old begins engaging in verbal and physical aggression (i.e., screaming and threatening their mother, throwing things at their mother). **OUTCOME:** Their mom says, “FINE! you’re going to do whatever you want anyway” and leaves the room. This 12-year-old is learning that their behavior (aggression) leads to the removal of perceived threat (i.e., *negative reinforcement*: mom blocking their goal) and increases the likelihood that they get to see the concert and connect socially (i.e., *positive reinforcement*). In the future, this means that aggression is more likely to occur the next time mom says “NO!”, and the next time they see mom with a glare, folded arms or speaking in a stern tone - even if she hasn’t said “NO!”.

Prior to reviewing the empirical literature in this area, we first offer an overview of stimulus-response and response-outcome contingencies and provide an example to demonstrate their role in eliciting (stimulus-response) and maintaining (response-outcome) aggressive behavior in youth (see Fig. 1). While a simplified summary of these specific learning contingencies is presented below, we acknowledge that these learning processes are complex and dynamic, occur in unison beyond our conscious awareness, and are continuously updated via ongoing interactions with the environment (Anderson & Bushman, 2002). We note that different functions of aggression (e.g., reactive versus proactive) likely vary in the extent to which they are under the control of stimulus-response vs. stimulus-outcome contingencies, and these processes vary between and within individuals and across interpersonal contexts, necessitating a person-centered approach to characterizing aggressive behavior.

### 2.1. Stimulus-response contingencies as antecedents of aggressive behavior

Examining stimulus-response contingencies allows us to identify individual- and dyad- level *antecedents* of aggressive behavior. A stimulus can be internal (e.g., negative emotion) and/or external (e.g., perceived threat) and precedes a specific behavioral response. The acquisition of these contingencies is examined within the context of classical conditioning (LeDoux, 2003; Pavlov, 1927), the process by which a stimulus of significance (unconditioned stimulus; UCS) is paired with a neutral stimulus (conditioned stimulus; CS), both of which come to elicit the same behavioral response (unconditioned response [UCR], conditioned response [CR], respectively). When examining aggression as the behavioral response, evaluating the stimuli (CS-UCS) that precedes aggressive behavior is necessary to proximally predict *when* it will occur. Here, aggression is conceptualized as a learned behavior that is (at least partly) under the control of an *antecedent* stimulus that functions to increase the probability of its occurrence (see Fig. 1).

### 2.2. Response-outcome contingencies as consequences of aggressive behavior

Response-outcome contingencies can be used to characterize individual- and dyad-level *consequences* of aggressive behavior. These consequences can be internal (e.g., reduction of negative emotion) and/or external (e.g., removal of perceived threat), and immediately follow a behavioral response. The acquisition of this contingency is assessed during operant conditioning, which refers to response contingent outcomes that increase and maintain behaviors (reinforcement) or decrease and extinguish behaviors (punishment) (Skinner, 1969). Contingent outcomes increase behavior via positive reinforcement (introducing positive stimuli) or negative reinforcement (removing aversive stimuli) and decrease behavior via positive punishment (introducing aversive stimuli) or negative punishment (removing positive stimuli). When examining aggression as the behavioral response, identifying reinforcing outcomes that immediately follow aggressive behavior is necessary to predict *why* it *re*occurs. Here, aggression is conceptualized as learned behavior that is (at least partly) under the control of contingent *consequences* that function to maintain and increase the likelihood of its *re*currence (see Fig. 1).

### 2.3. Summary of contingency learning theories & aggression

The contingencies that elicit and maintain aggressive behavior have been a central focus of etiological and intervention models of aggression in youth for decades (e.g., Blair, 2001; Blair, 2016; Patterson, 1982; Patterson, 2002). Better understanding individual differences in neurobiological sensitivity to these learning contingencies has the potential to further elucidate risk pathways for aggression. Historically, research examining neurobiological reactivity to stimulus-response-outcome contingencies has focused on *antecedents* and *consequence*s, with an emphasis on reactivity to individual-level external stimuli. Specifically, empirical work has examined perceived threat (e.g., provocation, goal-blocking, and frustrative non-reward) as *antecedent* stimuli that elicit aggressive behavior and the removal of perceived threat following aggression as a *consequence* that increases and maintains aggressive behavior (e.g., negative reinforcement via reducing provocation or positive reinforcement via obtaining a desired goal or reward). Theoretically, heightened neurobiological reactivity to proximal antecedents (e.g., perceived threat) and/or the reinforcing effects of proximal consequences (e.g., the removal of perceived threat) may independently or synergistically increase risk for aggression. Empirical work in this area has attempted to characterize neurobiological reactivity during contingency learning using paradigms that assess stimulus-response (*antecedents)* and response-outcome (*consequences)* contingencies. The subsequent sections provide a review of these findings.

## 3. Individual differences in neurobiological sensitivity during contingency learning

Research examining neurobiological reactivity to contingency learning has assessed individual differences in physiological reactivity and neural reactivity. Physiological indicators include 1) heart rate (HR), which measures autonomic function broadly, 2) skin conductance response (SCR) and eye-blink startle, which index sympathetic nervous system function, and/or 3) respiratory sinus arrhythmia (RSA), a measure of parasympathetic nervous system function. Neural reactivity has been assessed primarily via functional magnetic resonance imaging (fMRI). Below, we summarize research examining physiological and neural reactivity during stimulus-response (*antecedents*) and response-outcome (*consequences*) contingencies and point to empirical work that has examined these processes as risk factors for aggression in youth. Notably, work in this area has focused almost exclusively on associations with externalizing behaviors, broadly defined, with very few studies examinging aggression specifically.

### 3.1. Stimulus-response contingencies: Associations with aggressive behavior

Research examining stimulus-response contingencies has used a variety of classical conditioning paradigms to assess individual differences in sensitivity or reactivity to an aversive stimulus (UCS ➔ UCR) and a paired neutral cue (CS ➔ CR). Empirical studies have examined neurobiological reactivity during more traditional conditioning paradigms that assess reactivity to *uncued* (UCS: randomly presented aversive noise, noise burst) and *cued* (CS: neutral tone paired with UCS) experimental stimuli (see Byrd et al., 2014; Lorber, 2004 for reviews). More recent work has examined reactivity using alternative designs that assess reactivity to *uncued* stimuli (UCS) in the context of provocation or frustrative non-reward tasks as well as during retaliatory responses (UCR) to *uncued* aversive stimuli (see Bertsch et al., 2020; Fanti, 2018; Fanti et al., 2019 for reviews). We review this research below in two parts, focusing first on traditional conditioning paradigms and second on alternative paradigms that aim to enhance ecological validity.

#### 3.1.1. Traditional conditioning paradigms

We are aware of four studies that examined differences in physiological reactivity (i.e., skin conductance response) during more traditional conditioning paradigms, all of which were conducted by Gao, Raine, and colleagues. In their first longitudinal study of 200 children (50 % male; age range: 3–8 years), youth who showed reduced reactivity to a neutral cue (CS = non-aversive tone) paired with a loud noise burst (UCS) engaged in higher levels of aggression at age 8 (Gao et al., 2009). In a second longitudinal investigation (*n* = 278; 90 % male; age range: 10–18 years), youth demonstrating lower reactivity to a neutral cue (CS = non-aversive tone) paired with a picture of an attacking dog at age 18 reported higher levels of aggression, specifically proactive aggression (i.e., from ages 10 to 18 years; Gao et al., 2015). An additional study of children (*n* = 446; 52 % male; age range 11–12 years) showed bivariate associations between physiological reactivity to an *uncued* aversive stimulus (UCS = loud noise burst) and aggressive behavior (Chen et al., 2021).^1^ One final cross-sectional study of 340 children (48 % male, mean age = 9.06 years) failed to demonstrate associations between physiological reactivity during stimulus-response conditioning and aggression (Gao et al., 2018); however, this conditioning task included cued (CS = colored shape) monetary rewards and punishments (UCS), not an inherently aversive UCS. Overall, these findings mirror those from studies among youth with externalizing behaviors (broadly defined) whereby youth with externalizing behaviors demonstrate reduced physiological reactivity to *uncued* aversive stimuli (UCS; Fairchild et al., 2008; Herpertz et al., 2001; van Goozen et al., 2004), as well as reduced physiological reactivity to *cues* following pairing with aversive stimuli (CS; Fairchild et al., 2008; Loeber et al., 2007; Raine & Venables, 1981; Raine et al., 1996; Syngelaki et al., 2013).

Taken together, this work links *reduced* physiological reactivity to antecedent stimuli – *uncued* [UCS] and *cued* [CS] – with aggression in youth. It is important to note that many of these studies report no differences in physiological reactivity to aversive stimuli (UCS) *after* pairing with CS (i.e., differences in physiological reactivity were successfully transferred to the CS after pairing). Further, these studies report no differences in cognitive awareness of CS-UCS pairings (i.e., youth were able to identify which cue [CS] predicted an aversive stimulus [UCS]), suggesting that this reduction in physiological reactivity is *not* impairing CS-UCS learning under these conditions.

Historically, the findings described above have often been interpreted as evidence of punishment insensitivity, such that aggressive youth are less affected by punishment and less likely to learn from consequences (see Byrd et al., 2014). However, the studies reviewed here do not test sensitivity to punishment as *consequences* of aggression (i.e., response-outcome). Rather, they examine reactivity to *antecedent* stimuli, i.e., stimulus-response, which (could) precede aggression. While these findings could reflect an overall insensitivity to aversive environmental stimuli, such that aggressive youth would show reduced reactivity to aversive stimuli that precede (*antecedents*) *and* follow (*consequences*) aggression, it is important to note that these studies are not designed to test punishment sensitivity. This would require examining a change (or lack thereof) in behavior following punishment. Perhaps more importantly, these paradigms focus exclusively on reactivity to a “stimulus” (i.e., CS or UCS) that would be *unlikely* to elicit an aggressive response in the natural environment; thus, more ecologically valid studies are needed to examine individual differences in reactivity to stimulus-outcome contingencies within the context of aggression.

#### 3.1.2. Alternative conditioning paradigms

Recent work has shown some evidence of *increased* physiological and neural reactivity to stimulus-response contingencies with designs that have attempted to improve ecological validity, though aggression specific outcomes were again rare. While these studies have not examined stimulus-response contingency learning explicitly, they have examined reactivity to provocation and/or frustration, which can be conceptualized as *uncued* UCS, as well as reactivity during *uncued* retaliatory responses (UCR). These examinations are in line with theory suggesting perceived threat or frustration could serve as a proximal antecedent of retaliatory behavior like aggression (e.g., Berkowitz, 1993).

Several studies examined physiological reactivity during *uncued* provocation with a simulated peer using different laboratory tasks. In two samples of school-aged children (*n* = 272; mean age = 8; 100 % male; and *n* = 51; mean age = 10; 100 % male), increased HR and SCR reactivity following peer provocation was associated with teacher-reported aggression (Hubbard et al., 2002; Williams et al., 2003). Similarly, in a community sample of adolescents (*n* = 40: 100 % female; age range = 13–17 years), youth experiencing peer provocation (i.e., aversive noise bursts delivered by an opponent) showed greater HR reactivity and subsequently engaged in significantly more aggression toward their opponent (i.e., delivering aversive noise bursts to an opponent) relative to the control group (Rinnewitz et al., 2019). In a clinical sample of boys (*n* = 175; age range-9–13 years), boys with disruptive behavior disorders showed greater HR reactivity following low levels of provocation (i.e., loss of points) and engaged in higher levels of aggression (i.e., number of points participants taken from opponent) relative to healthy control youth (Waschbusch et al., 2002). However, two studies among samples of adolescents in a juvenile detention center (*n* = 85; 100 % male; age range = 13–18) showed no differences in physiological responding following provocation (Centifanti et al., 2013; Muñoz et al., 2008), suggesting no associations between physiological reactivity and the severity of aggression in high-risk youth.

One additional study examined RSA reactivity during a frustration task in 77 high-risk preschoolers (age range = 3–4 years; 56 % female; 47 % racial/ethnic minoritized status). Results showed that greater RSA reactivity (i.e., withdrawal) to frustration interacted with maternal invalidation to predict more teacher-reported aggression in a daycare or preschool setting (Byrd et al., 2023). This echoes recent work linking greater RSA reactivity (i.e., withdrawal) during parent-child conflict to aggressive behavior among high-risk adolescents (Byrd et al., 2022). Moreover, these findings are in line with research demonstrating associations between increased physiological reactivity during frustration tasks and externalizing behaviors (broadly defined) assessed cross-sectionally (Degnan et al., 2008; Gatzke-Kopp et al., 2015) and longitudinally (El-Sheikh et al., 2011; Erath et al., 2011).

Research examining neural reactivity during similar provocation paradigms is restricted to clinical samples. In a clinical sample of boys (*n* = 36; age range = 9–14 years), youth with disruptive behavior disorders showed decreased activation in the ventral anterior cingulate cortex and the temporoparietal junction during aggressive responses to provocation relative to a control group (i.e., removing points from an opponent; Bubenzer-Busch et al., 2016). In another clinical sample of youth (*n* = 56; age range = 10–18 years, 41 % female), those with disruptive behavior disorders demonstrated increases in activation of basic threat circuitry (i.e., amygdala, periaqueductal gray) and reduced activation in the ventromedial prefrontal cortex during retaliation (White, VanTieghem, et al., 2016). Finally, adolescents in residential care who displayed aggressive behavior (*n* = 42; age range = 13–18; 39 % female) were also characterized by reduced activation in regions associated with expected value (i.e., ventromedial prefrontal cortex and left posterior cingulate cortex) and inhibitory control (i.e., inferior frontal gyrus and bilateral anterior insular cortex) when receiving an unfair offer and/or engaging in retaliation (Mathur et al., 2023).

These findings are in line with recent meta-analytic work in healthy populations which show *reduced* activation during *uncued* frustrative non-reward in brain regions implicated in detecting salient environmental stimuli (e.g., orbitofrontal cortex, ventral striatum, and posterior cingulate cortex) coupled with *increased* activation in the bilateral frontoinsular cortex, dorsal anterior cingulate cortex, and anterior midcingulate cortex (Dugré & Potvin, 2023). Interestingly, there was significant overlap in neural activation during retaliatory responses that followed *uncued* ‘provocation’ (i.e., punish or reject an unfair offer), with the fronto-insular and midcingulate cortices demonstrating activation during both *uncued* frustrative non-reward *and* retaliatory responses. Taken together, these findings highlight aberrant neurobiological reactivity during stimulus-response learning may contribute to the development and persistence of aggressive behavior. However, notable differences in directionally (increased versus decreased reactivity), variability in task designs, and paucity of studies that focus specifically on aggression underscore the need for continued research in this area.

#### 3.2. Response-outcome contingencies: Associations with aggressive behavior

Studies designed to test response-outcome contingencies have utilized various operant conditioning paradigms (e.g., response reversal learning, passive avoidance learning) to assess individual differences in the sensitivity to response contingent outcomes and the extent to which this changes behavior. Research in this area has assessed neural activation to response contingent reinforcing and punishing outcomes as well as behavioral changes in responses following reinforcing and/or punishing outcomes; however, we are aware of no studies that examine associations with aggression specifically. Those studies that have examined response-outcome contingencies focus on externalizing behavior, broadly defined, and almost exclusively utilize neuroimaging to examine neurobiological reactivity.

Within the neuroimaging literature, research has shown that youth with externalizing behavior evidence reduced reactivity in the ventromedial prefrontal cortex, anterior cingulate cortex, and insula during the processing of response-outcome contingencies (for reviews see Alegria et al., 2016; Blair et al., 2018). Much of this research has utilized learning paradigms to examine reinforcement-based decision-making, with recent findings showing aberrant neural reactivity to prediction errors and expected value signaling (e.g., Finger et al., 2011; White et al., 2013; White, Tyler, et al., 2016). There is also a considerable body of work examining neural reactivity to the receipt of reward and punishment outcomes, most often monetary gain versus monetary loss. There is some evidence to suggest that youth with externalizing behavior show greater activation to the receipt of reward versus the receipt of punishment (e.g., Byrd et al., 2018; Byrd et al., 2021; Hawes et al., 2021); however, reactivity to these outcomes were assessed outside of the context of learning. Thus, while these studies certainly inform our understanding of contingency learning (Lutz & Widmer, 2014), more focused work on stimulus-outcome learning as a risk factor for aggression specifically is needed.

#### 3.3. Summary of individual differences in neurobiological sensitivity during contingency learning

Research on examining neurobiological reactivity during contingency learning is notably sparse among youth with aggression. Only a handful of studies examined reactivity to stimulus-response contingencies as a predictor of aggression, and no studies have assessed reactivity to response-outcome contingencies as a predictor of aggression specifically. While this work suggests potential differences in neurobiological sensitivity during the processing of the learning contingencies, research has focused primarily on samples of youth with externalizing behaviors, broadly defined, which drastically limits our understanding of the neurobiological underpinnings of aggression. This is surprising given how central stimulus-response-outcome contingencies are to etiological and intervention models of aggression in youth (e.g., Blair, 2001; Blair, 2016; Patterson, 1982; Patterson, 2002). As research moves forward in this area, it will be imperative that we examine individual differences in neurobiological sensitivity during contingency learning as predictors of aggression specifically to enhance our understanding of how proximal antecedents may elicit aggression and immediate consequences maintain problem behaviors.

## 4. Next steps: Conceptual and methodological considerations

While contingency learning theories emphasize the dynamic, momentary processes that elicit and maintain aggression, the research summarized above examined individual differences in sensitivity to stimulus-response-outcome contingencies as static, between-person risk factors that are used to examine distal associations with aggression. Moreover, empirical work in this area has focused most prominently on externalizing behaviors, broadly defined. As such, shifting our examination of learning contingencies to more accurately assess dynamic, within-individual and within-dyad processes as momentary predictors of aggression specifically is critical to enhancing our ability to proximally predict *when* an individual will engage in aggression (antecedents) and *why* they continue to do so (consequences). Given that the meaning or valence of these contingencies vary between- and within-individuals, with noted variability across interpersonal (e.g., one caregiver vs. another caregiver) and environmental (e.g., home vs. school) contexts, it is important that we shift our approach to obtain a more precise, individually tailored mapping of the ‘contingency landscape’. Enhancing the ecological validity of our study designs would further our understanding of how differences in neurobiological sensitivity contribute to within-individual and within-dyad processes resulting in aggression. Ultimately, this would enable those who hope to reduce aggressive behavior to formulate a personalized intervention plan that could be calibrated to contingency processing biases and social contexts. In the following sections, we advocate for a more precise characterization of proximal stimulus-response-outcome contingencies as a means for enhancing our ability to predict *when* aggression will occur and *why* it continues and offer specific recommendations for conceptual and methodological advancements of this research.

### 4.1. Conceptual considerations: Expanding the framework to enhance contingency learning models of aggression

Prior research on neurobiological sensitivity during contingency learning has undoubtedly enhanced our understanding of the development and persistence of aggression. However, as highlighted above, this research has been limited in several ways. Below, we describe how expanding our conceptual framework to 1) examine aggression transdiagnostically and 2) consider alternative antecedents and consequences of aggression has the potential to enhance our understanding of aggressive behavior.

#### 4.1.1. Aggression as a transdiagnostic construct

Although aggression permeates nearly all psychiatric disorders in youth (Kazdin, 2003) and represents the most common reason youth are referred for mental health treatment (Coccaro & McCloskey, 2018; Sukhodolsky et al., 2016), examinations of aggression have often been limited to disorder-specific samples (e.g., externalizing disorders). Along these lines, empirical work examining individual differences in neurobiological sensitivity to contingency learning is largely based on research that has focused on externalizing behaviors, broadly defined. While these studies have provided evidence for aberrant physiological and neural processing among children who are vulnerable to externalizing disorders, this approach has slowed our understanding of the neurobiological underpinnings of aggressive behavior specifically. Additionally, it has impeded the progression of our understanding of aggression as it exists across other conditions and precluded the advancement of a transdiagnostic mechanistic model of aggression. This circumscribed focus on externalizing disorders as a means for understanding aggression is longstanding. However, aggression is prominent among youth with externalizing disorders (i.e., oppositional defiant disorder [ODD], conduct disorder [CD]), as well as those with neurodevelopmental disorders (i.e., attention deficit hyperactivity disorder [ADHD] and autism spectrum disorder [ASD]) and internalizing disorders (i.e., depression, anxiety), underscoring the prevalence and clinical relevance of aggression across the diagnostic spectrum (Coccaro & McCloskey, 2018). Thus, to advance our understanding of neurobiological sensitivity during contingency learning in aggressive youth, future work should sharpen our focus to assess the functions of aggressive behavior specifically while widening our lens to include aggression regardless of diagnostic categories.

#### 4.1.2. Considering alternative antecedents and consequences of aggression

**Fig. 2.**
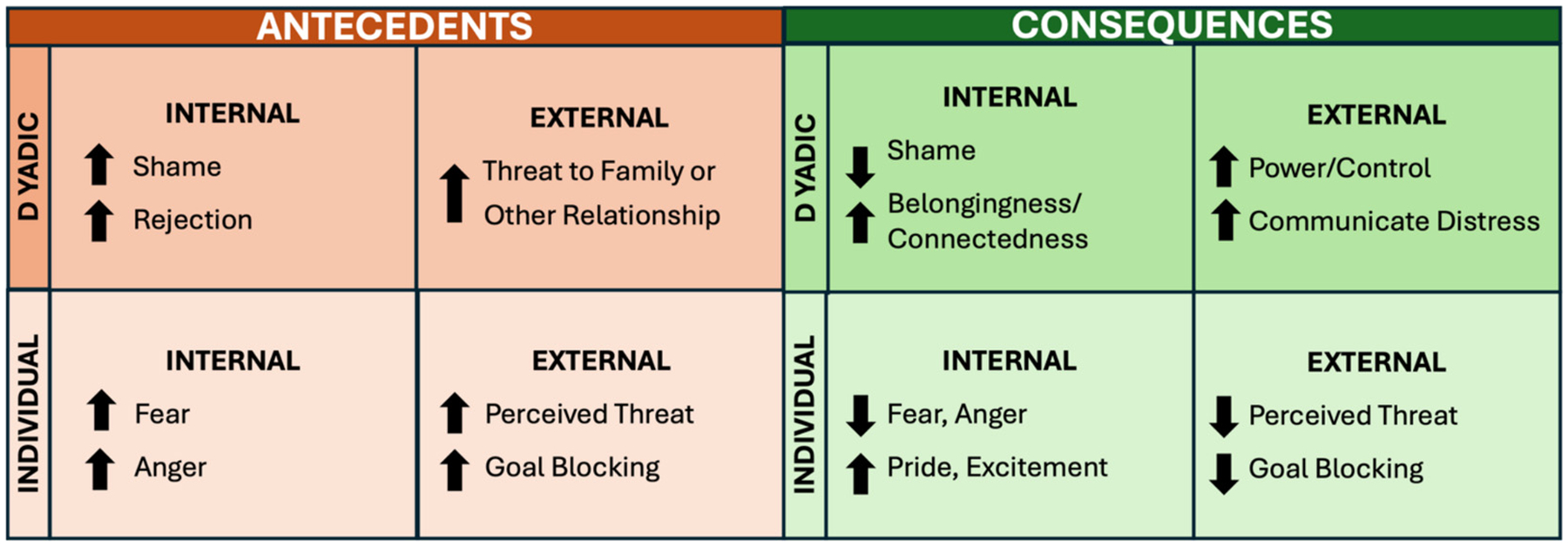
Internal and external antecedents and consequences of aggression are depicted at the individual- and dyadic level. Arrows indicate an increases (up arrow) or decreases (down arrow).

While contingency learning theories of aggression point to a variety of internal and external antecedents and consequences that elicit and maintain aggressive behavior in youth, empirical work examining neurobiological reactivity has focused almost exclusively on individual-level *external* antecedents and consequences (i.e., stimuli and outcomes encountered in the surrounding environment). Specifically, studies have utilized *external* antecedent stimuli that signal perceived threat (e.g., provocation) or frustrative goal blocking and assess the removal of perceived threat and/or attainment of a desired goal immediately following aggression as *external* consequences. An examination of physiological and neural processing during stimulus-outcome contingencies that focus on individual-level *internal* antecedents and consequences of aggression independent from or in combination with *external* antecedents and consequences is essential for advancing etiological and intervention models of aggression. This includes *internal* antecedents, such as increased negative affect (e.g., anger, fear), and *internal* consequences, such as decreases in negative affect and/or increased positive affect (e.g., pride, excitement) that occur immediately following aggressive behavior (see Fig. 2).

In addition to expanding our conceptualization and empirical examination of individual-level internal *and* external antecedents and consequences of aggression, there is a strong need to incorporate the interpersonal context from which aggressive behavior emanates. Unsurprisingly, many theories of aggression explicitly underscore the importance of relationship factors. For example, the adult literature highlights critical dyadic processes that perpetuate intimate partner violence (Cochran et al., 2017; Powers et al., 2020), and prominent theories of aggression in youth detail transactional dyadic parent-child processes that elicit and maintain aggressive behavior (Patterson, 1982; Patterson, 2002; Scaramella & Leve, 2004). However, empirical work examining the neurobiological underpinnings of aggression in youth has focused almost exclusively on the aggressive individual and failed to consider the dyadic context in which the aggressive individual exists. Shifting our focus to potent dyadic, interpersonal mechanisms of aggression will further enhance the prediction of *when* aggression will occur and *why* it continues. These interpersonal antecedents and consequences may include dyad-level *internal* antecedents such as increases in social emotions (e.g., rejection, embarrassment) and dyad-level *internal* consequences, such as decreases in rejection or embarrassment and/or increases in positive social emotions like sense of belonging and/or connectedness to others following aggression. Similarly, dyad-level *external* antecedents, such as threats to family or the relationship, and dyad-level *external* consequences, such as asserting power or control over others or communicating feelings of distress are also worthy of consideration (see Fig. 2).

Importantly, our characterization of neurobiological sensitivity to the internal and external antecedents and consequences of aggression requires a shift from examining these contingencies as static, distal, between-person risk factors to characterizing the dynamic, proximal processes that occur within the individual and the dyad. This necessitates expanding our study designs to allow for an assessment of neurobiological reactivity to these contingencies as time-varying mechanisms that unfold within interpersonal interactions in real time. Study designs assessing dynamic processes that unfold over time also require more advanced statistical approaches that can accommodate the complex nature of intensive repeated assessment data and allow for the testing of hypothesized mechanisms. Ultimately, these advancements will increase the precision with which we are able to map the ‘contingency landscape’ within-individuals and within-dyads, further refining etiological models of aggression and identifying more personalized targets for intervention.

### 4.2. Methodological Considerations: Alternative study designs to enhance ecological validity

Given that prominent theoretical models of aggression emphasize the importance of characterizing momentary, dynamic changes in within-individual and within-dyad processes, expanding our assessment of neurobiological reactivity to the antecedents and consequences of aggressive behavior within this framework is essential. Here, we describe how experimental laboratory paradigms may be enhanced to achieve these aims and the potential utility of incorporating widely used ambulatory assessment methods in aggression research.

#### 4.2.1. Developing alternative laboratory paradigms

Laboratory paradigms assessing neurobiological reactivity during contingency learning often utilize standardized protocols with high experimental control. However, there are notable limitations to ecological validity that are particularly relevant when thinking about enhanced prediction of aggression. Research in this area has almost exclusively tested learning models of aggression using laboratory paradigms that are 1) void of aggression and 2) define aggression as a static event that emanates from the individual (e.g., number of button presses as a representation of aggressive behavior) following a contrived antecedent within manufactured interpersonal interactions (e.g., imaginary fictional character taking “money”;McCarthy & Elson, 2018; Tedeschi & Quigley, 1996). Importantly, there are few, if any, consequences for engaging in aggression (e.g., loss of “money”) and these consequences are rarely examined as potential maintenance factors for future aggression. While these tasks offer high experimental control and can be beneficial for isolating and testing specific components of stimulus-response-outcome contingencies, there is increasing need to develop alternative laboratory tasks that improve our ability to assess neurobiological reactivity to proximal internal *and* external antecedents and consequences of aggressive behavior as dynamic processes that occur within individuals *and* dyads.

As described above, more recent research has attempted to enhance ecological validity by including tasks that introduce antecedents that are common prompting events for aggression (e.g., provocation, frustrative goal blocking) and measuring interpersonal behavioral responses that approximate aggression. In these tasks, the type of provocation (e.g., verbal versus physical provocation) can be experimentally manipulated to closely study how specific kinds of provocation differentially evoke behavioral responses like aggression (Jones & Paulhus, 2010). Enhancing these paradigms to include continuous multimodal assessment of neurobiological reactivity to characterize within-individual changes during stimulus-response *and* response-outcome contingency learning would provide essential information about the dynamic time-course of these processes. In particular, paradigms that assess the consequences of aggressive behavior are needed to understand the maintenance of aggression over time (e.g., examining within-individual changes in neurobiological reactivity before *and* after engaging in aggressive behavior). This might include using virtual reality to generate a more realistic assessment of stimulus-response-outcome contingencies (Wilson & Soranzo, 2015), and could further increase external validity by incorporating avatars to mimic interpersonal interactions (see Lobbestael & Cima, 2021; Terbeck et al., 2022). Additionally, including parents or peers in laboratory protocols (e.g., conflict or rejection paradigms) could allow for an examination of contingency learning within dyadic interactions, further enhancing external validity. While these advancements could allow for important opportunities to characterize neurobiological reactivity as within-individual and within-dyad processes, all laboratory assessments of aggression are limited by demand characteristics and ethical constraints that preclude reliable elicitation of aggressive behavior (McCarthy & Elson, 2018; Tedeschi & Quigley, 1996). Thus, incorporating study designs that aim to capture the neurobiological underpinnings of contingency learning as they spontaneously occur in daily life has the potential to provide important information about what precipitates aggression and what contributes to its maintenance and escalation over time.

#### 4.2.2. Incorporating assessments of aggression in daily life

Ambulatory assessment study designs are well-suited to assess neurobiological reactivity during the proximal antecedents and consequences of aggressive behavior as they unfold (over minutes, hours, days) within a naturalistic context. These designs aim to capture phenomena like aggression as it spontaneously occurs ‘in the wild’ and offer significant advantages over attempted lab-based inductions of low base rate behaviors like aggression and/or retrospective report of the proximal antecedents and consequences of aggression (Shiffman et al., 2008).

Ambulatory assessments include a variety of data collection methods focused on capturing momentary, dynamic interactions in daily life (Trull & Ebner-Priemer, 2013). These protocols are well known for incorporating brief surveys (e.g., less than 5 min) delivered via smartphones at random and/or event-contingent times throughout the day to assess emotion and behaviors. Emerging work also includes continuous passive sensing of neurobiological reactivity (e.g., heart rate, RSA) as well as other important indicators, like communication via audio recording or text analysis of smartphone data, and/or location (e.g., proximity to certain people or places). Within-individual changes in physiological reactivity have been utilized to trigger audio recordings and surveys in an event-contingent way, e.g., changes in physiology as an indicator of a potential stress (de Geus & Gevonden, 2023), and/or proximity to a parent or peer (Boateng et al., 2022). This has potential utility for increasing the likelihood of capturing aggressive behavior during its occurrence ‘in the wild’. Additionally, these multimodal indicators (e.g., physiology, proximity) can be analyzed alongside survey and audio data to characterize proximal antecedents and consequences of aggression.

Importantly, ambulatory protocols can include both the individual (youth) and other members of a dyad (e.g., parent or peer) to allow for a continuous assessment of neurobiological reactivity within individuals *and* within dyads, enhancing our ability to predict *when* aggression will occur and *why* it continues. Specifically, this methodology can capture proximal antecedents and consequences of aggression at different temporal resolutions (e.g., seconds, minutes, hours, days) within more naturalistic contexts, and are thus well-positioned to test personalized contingency learning models of aggression within interpersonal interactions (Collins, 2013). However, despite strong correspondence between contingency learning models of aggression and these dynamic assessment approaches, research in this area has seen only a modest uptake of ambulatory assessment designs (e.g., Colasante et al., 2016). Additionally, incorporating these more naturalistic assessments necessitates advanced analytic approaches that can handle intensive longitudinal data and more closely adhere to the complex and dynamic system of predictors and outcomes involved in contingency learning models of aggression.

### 4.3. Methodological considerations: Advanced analytic approaches

The use of advanced analytic approaches (e.g., computational modeling, machine learning, dynamic structural equation modeling) are ideal and necessary for examining proximal antecedents and consequences of aggressive behavior as momentary, dynamic processes within-individuals and within-dyads. These analytic techniques allow us to empirically test contingency learning models of aggression and significantly enhance our ability to predict *when* aggression will occur and *why* it continues. Below we summarize the potential benefits of incorporating computational, machine learning, and dyadic structural equation modeling approaches and point to emerging literature that has capitalized on these methods within aggression research.

#### 4.3.1. Computational modeling & machine learning approaches

In the past decade, computational modeling in mental health research has seen a renewed interest given long-standing theoretical frameworks that take a dynamic systems approach to mental health (e.g., network theory; Borsboom, 2017). These models focus specifically on identifying complex, mechanistic processes that give rise to behaviors like aggression and aim to predict future aggressive behavior. Computational modeling quantifies a theoretical or conceptual model through simulation-based analyses, which rely on differential equations to describe the rate of change in each variable (e.g., physiological or neural reactivity) as a function of itself and other causally related variables (e.g., provocation).^2^ In a computational model, every component in a system can be expressed as a differential equation, and the system being modeled can be tailored to a specific individual or dyad (Lockwood & Klein-Flügge, 2021; Pauli & Lockwood, 2023). Indeed, emerging research on aggression has utilized computational modeling when examining the underlying neurobiological reactivity during contingency learning as a risk factor for aggressive behavior (e.g., Elster et al., 2024; White et al., 2013). For aggression researchers, the primary benefit of computational modeling is its formalization of how variables change over time (both in relation to themselves and other variables) and thus can serve as a particularly valuable tool for advancing contingency learning models of aggression.

Machine learning models have also become increasingly popular in clinical research given their ability to optimize the prediction of behavior using all available data (Chekroud et al., 2021; Dwyer et al., 2018). Importantly, these models can also incorporate a large number of predictors derived from multiple modalities (Dwyer et al., 2018), and when combined with Bayesian modeling, have the ability to incorporate prior knowledge (Van De Schoot et al., 2017). These techniques are especially useful for modeling complex, dynamic interactions among many variables, which has clear relevance for testing contingency learning models of aggression. By taking a bottom-up approach, these models can be used to identify commonalities in dynamic neurobiological processes as well as nuances specific to individuals (e.g., Jacobson & Chung, 2020). This can provide an empirical characterization of the degree of heterogeneity in reactivity to internal and external antecedents and consequences that proximally predict aggressive behavior, as well as identification of individual-specific momentary processes that will serve as high-value treatment targets to include in personalized interventions for aggression (Goodwin et al., 2020). While we want to avoid overemphasizing the distinction between machine learning and other statistical techniques (Finlayson et al., 2019), we note that machine learning prioritizes out-of-sample prediction (Yarkoni & Westfall, 2017), in which thedata used to test the predictive accuracy of a model is independent from the data used to develop or train the model. In some research contexts, out-of-sample prediction accuracy may be of significant value to aggression researchers (e.g., developing predictive models focused on when someone will act aggressively).

To date, research has primarily used computational modeling and machine learning to examine high-dimensional data derived from ambulatory assessment that include wearable devices (e.g., physiological data from biosensors). For example, Imbiriba and colleagues (2023) examined whether data derived from a biosensor could be used to construct predictive models of aggression, along with emotion dysregulation and self-harming behavior, in a sample of psychiatric inpatient youth. When collapsing across instances of emotion and behavior dysregulation, accurate prediction three minutes prior to onset had an AUC^3^ of 0.80; with models that focused specifically on aggressive behavior showing slightly lower predictive accuracy (AUC range = 0.70–0.80). In another study, Park and colleagues (2023) utilized data obtained from waist-worn biosensors in combination with demographic (e.g., age) and symptom scores to predict youth aggression during a one-week ambulatory assessment period. The authors were able to accurately predict the onset of physical aggression (AUC = 0.87) and found that the most important predictors were biosensor-assessed physical acceleration (i.e., faster triaxial acceleration), child age (i.e., younger children being likely to engage in aggression), and total symptom scores. While inclusion of computational modeling and machine learning in aggression research is in its infancy, there is notable utility in advancing our statistical approaches alongside conceptual and methodological expansions as they maximize our ability to empirically test more complex theoretical models, enhancing our ability to predict aggressive behavior.

#### 4.3.2. Dynamic structural equation modeling

The ability to model proximal changes in neurobiological reactivity to the antecedents and consequences of aggressive behavior as a dynamic process that occurs *within the dyadic context* requires advanced quantitative methods to model intensive dyadic longitudinal data (Asparouhov et al., 2018; Hamaker et al., 2018). Dynamic structural equation modeling (DSEM) is at the cutting-edge as it combines the respective strengths of time series analysis (specifically modeling autoregressive or lagged effects), multilevel modeling, and structural equation modeling, and has important advantages over traditional multilevel models (McNeish & Hamaker, 2020). First, these models incorporate between-person differences (e.g., random slopes) in within-individual *and* within-dyad associations and these can be used as both predictors and outcomes, allowing for an examination of within-individual and within-dyad processes. Additionally, these models can incorporate multiple predictors and outcomes simultaneously, allowing for an examination of the independent and synergistic effects of multiple internal and external antecedents and consequences of aggression. Finally, the ability to construct latent variables at each level of the model allows for researchers to combine information across multiple modalities and informants while accounting for measurement error in observed variables.

The application of DSEM to intensive dyadic longitudinal data can be implemented to optimally utilize data collected during laboratory and/or ambulatory protocols with dyads (e.g., parent-child) and characterize momentary, dynamic changes in proximal predictors of aggression. While we refer readers interested in the finer details of DSEM and similar methods to previously published primers (McNeish & Hamaker, 2020; Sadikaj et al., 2021),^4^ we briefly highlight how modeling approaches like DSEM can enhance learning models of aggression. For example, a typical model involving youth and their parent would include the lagged relations between youth physiological reactivity, subjective negative affect, and aggression at time *t − 1*, time *t,* time *t* + *1*, along with the same lagged effect for parent behavior. Including cross-lagged associations can test whether youth physiological reactivity and/or subjective negative affect from a previous timepoint is linked to youth aggression at a subsequent timepoint (i.e., changes in physiological reactivity or subjective negative affect as an internal antecedent of aggressive behavior). It can also test whether changes in physiological reactivity or subjective negative affect, or parent behavior following youth aggression predict the reoccurrence of aggression at a subsequent timepoint. Thus, aggressive behavior is an individual (level-1) variable, nested within dyads (level-2). Random effects can be included to examine between-dyad differences in the strength of effects, as well as modeling random effects for the residual variance of aggression (i.e., the variance in daily aggression not accounted for by lagged and cross-lagged effects). Incorporating these random effects would allow for the examination of the “volatility” of aggression within and between dyads and can help us better understand whether dyads differ from each other in how accurately aggression is predicted across each day. Thus, in contrast to prediction in machine learning approaches which is focused on out-of-sample prediction, DSEM is typically focused on predicting relevant within-person and within-dyad behaviors and outcomes from one timepoint to the next (i.e., autoregressive and cross-lagged effects), with the strength of prediction measured by regression coefficients.

In a recent application of DSEM, Brown et al. (2023) examined how strongly provocation predicted aggression and how engaging in aggression predicted the reoccurrence of aggression among a sample of youth with ADHD. Results showed significant autoregressive effects for both provocation and aggression such that youth who reported greater aggression or provocation relative to their own mean tended to report more feelings of provocation and aggression at the next assessment, and the carry-over effect for aggression was even stronger for youth reporting a higher number of ADHD symptoms. Notably, these effects were characterized using standardized regression coefficients and ranged between 0.09 and 0.15 which represent small effects. It will be important for future work to explore how well DSEM results at the sample level generalize to individuals within the same sample. For example, in most intensive longitudinal studies, it is common to observe significant variability in the magnitude of regression associations among variables (i.e., variance estimates of random slopes are often statistically significantly greater than zero). This underscores significant differences in the strength of associations (i.e., predictive power) within a sample. It is extremely likely that similar variability will be found when modeling the antecedents and consequences of aggressive behavior, with some youth demonstrating stimulus-response-outcome contingencies that are more predictable than other youth. DSEM models allow for this to be directly explored by reporting results at both the sample-level *and* individual/dyad-level (for examples of using DSEM in this way, see Rodebaugh et al., 2023; Vize et al., 2024).^5^ Ultimately, DSEM provides a valuable framework for expanding our assessment of contingency learning to include within-dyad process (e.g., impact of changes in parent behavior on child affect or behavior), while also examining how within-individual changes in neurobiological reactivity or subjective negative affect may exacerbate or dampen these associations over time, thereby increasing or decreasing aggression.

### 4.4. Expanding conceptually and methodologically to enhance clinical utility

Expanding our conceptual framework and advancing our methodological approaches when examining contingency learning and aggression has direct implications for intervention models of aggression. Specifically, contingency learning models are central tenets of Cognitive-Behavioral Therapy (CBT) and Parent Management Training (PMT), two of the primary interventions for aggression in youth (Kazdin, 2003; Kendall, 1991). These gold standard interventions closely align with the empirical literature reviewed here and often focus on individual-level antecedents (e.g., youth have distorted thoughts about perceived threat) and consequences (e.g., youth ‘get what they want’ following aggressive behavior). For example, CBT aims to reduce risk for aggression by helping youth to identify and change their (distorted) thoughts about perceived threat while introducing alternative behavioral responses to reduce risk for engaging in aggressive behaviors (Sukhodolsky et al., 2016). PMT, which is notably more dyadic in nature, helps parents to not ‘give in’ to their child’s escalation and instead implement consistent and immediate consequences following aggression (e.g. timeout, loss of privileges) while also reinforcing more desirable behaviors (Kazdin, 2008). While these treatments have been shown to be effective for some youth and their families, they demonstrate small-to-moderate effect sizes at best (Michelson et al., 2013; Smeets et al., 2015; Sukhodolsky et al., 2004), meaning many youth and families with many youth deemed “treatment-resistant”.

While there are number of broader, systemic factors that undoubtedly influence the effectiveness of these interventions (see Additional Considerations), it is also true that ‘one-size-fits all’ protocol-driven treatments are more limited in their ability to individually tailor intervention strategies for aggressive youth and their families. In contrast, principle-oriented treatments, like dialectical behavior therapy (DBT; Linehan, 2014) and functional behavioral analysis (ABA; Hanley et al., 2003; Haynes & O’Brien, 1990), focus specifically on the momentary assessment of individual *and* dyad-level antecedents and consequences (i.e., stimulus-response-outcome) that elicit and maintain problematic or ineffective behaviors across contexts. DBT was originally developed for the treatment of chronically suicidal adults with severe emotion dysregulation (Linehan, 1993), and has since been adapted for youth with similar problems with dysregulation (MacPherson et al., 2013), while ABA has been most commonly implemented among youth with autism spectrum disorders and other neurodevelopmental disabilities (Lovaas, 1987; Peters-Scheffer et al., 2011). Both interventions align closely with contingency learning models and require an initial comprehensive assessment to characterize internal *and* external antecedents and consequences at the individual and dyad level that inform individually tailored intervention strategies. These interventions include an ongoing assessment of problem behaviors, often via direct observation (e.g., in-office, phone coaching) and behavioral chain analysis, and intervention strategies are modified accordingly. Such approaches aim to identify and target antecedents via changing meaning or valence of internal or external stimuli and/or changing the probability of a behavioral response via changing contingent outcomes and implementing alternative behaviors. Importantly, more recent applications of these principle-oriented interventions have shown promise with youth engaging in aggression (Ciesinski et al., 2022; Frazier & Vela, 2014; Jakubovic & Drabick, 2023) and their families (Zalewski et al., 2018; Zalewski et al., 2023), highlighting their potential for implementation with aggressive youth.

Other examples of dynamic, momentary, evidence-based interventions include Parent-Child Interaction Therapy (PCIT; Eyberg et al., 2001) and Multisystemic Therapy (MST; Henggeler et al., 1986; Henggeler et al., 2009), both of which were developed for aggressive behavior in youth. PCIT focuses on aggression in early childhood and parents are observed during naturalistic interactions with their children while being live coached via earpieces. This coaching focuses primarily on response-outcome contingencies and aims to scaffold parents to more effectively respond to their child’s behavior using behavioral management strategies and positive parenting skills (Eyberg et al., 2001). This therapy has been found to be efficacious for treating aggression in children (Ward et al., 2016) and increasing parenting efficacy (Bjørseth & Wichstrøm, 2016). Moreover, recent adaptations of PCIT focus specifically on enhancing emotional responses and empathy in those most at-risk for persistent aggression (e.g., Kimonis et al., 2019; Kimonis & Armstrong, 2012). MST was designed for adolescents with more serious delinquency. This intervention also aims to address learning contingencies and expands the transactional focus to address multiple systemic levels, including the individual, family, peers, school, and neighborhood via in-home sessions and round-the-clock therapist support (Van der Stouwe et al., 2014). While MST was initially developed to help youth desist from delinquency (Henggeler et al., 1986), evidence suggests that MST is helpful for preventing not only delinquency but also onset of psychopathology, substance use, out-of-home placement, and improving family and peer factors (Curtis et al., 2004; Van der Stouwe et al., 2014). Despite the success of these interventions, they are more resource- and cost-intensive, and because of this, utilized less frequently than CBT and PMT. They may, however, be more cost-effective for youth who are not showing improvement with more traditional interventions and/or have increased risk for inpatient or residential placements. As empirical work on the neurobiological sensitivity to the contingencies that elicit and maintain aggression advances conceptually and methodologically, so does our ability to better characterize risk mechanisms and identify who may benefit most from these more intensive, process-oriented interventions.

Along these lines, our advanced modeling approaches (e.g., machine learning models, DSEM) allow for more enhanced prediction of aggressive behavior in daily life which raises important considerations for how results from this work could guide intervention efforts. For example, passive sensing could be used to identify personalized risk indicators of aggression (e.g., significant within-individual changes in physiological reactivity coupled with proximity to a parent or peer). These indicators could then be used to trigger the delivery of just-in-time interventions designed to provide high-quality, empirically-supported behavioral strategies to youth and/or their parents in real time. Importantly, we note that ‘perfect prediction’ is not possible nor is it necessary (Domingos, 2012), as these models aim to use prior data to optimize the prediction of critical moments for intervention when aggression would be most likely (Nahum-Shani et al., 2018). Notably, a growing literature has highlighted important challenges in developing and implementing effective digital mental health interventions like these (see Borghouts et al., 2021), with a common challenge being retaining engagement. Specifically, evidence suggests that only a very small number of individuals maintain a consistent level of engagement with digital mental health applications (Baumel et al., 2019). Thus, development of just-in-time interventions for aggression would need to include direct feedback from youth and their families, as an important predictor of engagement is whether the intervention is customizable and directly relevant to the user (Baumel et al., 2019). While this is a labor-intensive undertaking, past work has consistently documented that a participant-centered approach can effectively increase engagement and commitment, ultimately enhancing the effectiveness of these interventions (e.g., Bos et al., 2022; Lyon et al., 2020).

### 4.5. Additional considerations

While an examination of enhancing contingency learning theories of aggression requires a focus on the ‘local’ environment (e.g., individual- and dyad-level) within which aggression occurs, there are important additional considerations that exist across multiple systems that are noteworthy. For example, shared genetic vulnerabilities could underpin differences in how parent-child dyads attend to and process learning contingencies that elicit and maintain aggression (Viding et al., 2023). In the case where children are growing up with their biological families, parents may share similar neurobiological sensitivities to contingency learning, which may result in mutual challenges that are particularly likely to escalate conflict within dyadic interactions and increase risk for aggression (Viding & McCrory, 2012). Further, it is important to acknowledge that dyadic systems are embedded within broader familial, societal, and historical systems that exert transactional influences on youth across development (Bronfenbrenner, 1979; Viding et al., 2023). That is, neurobiological reactivity to antecedents and consequences is influenced by the broader systems of which they are a part, and our work in this area should also seek to incorporate an examination of these influences to further enhance our understanding of risk and resilience processes.

## 5. Conclusion

Contingency learning theories offer a valuable framework for identifying the *antecedents* (stimulus-response) and *consequences* (response-outcome) of aggression, and prominent etiological and intervention models of aggression are grounded in this work. Although decades of research have examined individual differences in sensitivity to stimulus-response-outcome contingencies, the majority of work in this area has focused on externalizing behavior broadly defined, leaving unanswered questions about the neurobiological underpinnings of aggression. Furthermore, these studies have typically examined neurobiological reactivity to individual-level factors (e.g., perceived threat) as a static, distal, between-person risk factor rather than a dynamic, proximal, within-person or within-dyad process. Our limited understanding of momentary changes in neurobiological reactivity to individual- and dyad-level contingencies that elicit and maintain aggression has drastically limited our ability to proximally predict *when* an individual will engage in aggression (antecedents) and *why* they continue to do so (consequences), ultimately hindering our ability to personalize interventions to maximize effectiveness.

Above we offer specific recommendations for conceptual and methodological expansions of this research with an eye toward optimizing the precision of intervention efforts. Specifically, we recommend that future research sharpen its focus to assess aggression specifically while also widening its conceptual lens to include transdiagnostic presentations as this is necessary to advance a comprehensive mechanistic model of aggression. In addition to the typically-explored individual-level *external* antecedents and consequences (e.g., perceived threat, goal blocking), we encourage researchers to consider alternative *internal* and *external* antecedents and consequences that elicit and maintain aggression while also considering *dyad-level* contingencies that give rise to and exacerbate aggression. Methodologically, advancements in this area will require innovative laboratory paradigms that allow for assessment of within-individual and within-dyad processes as predictors of aggression and leverage well-established ambulatory assessment methods to capture the unfolding of these processes as proximal antecedents and consequences of aggression in more naturalistic environments. This work necessitates advanced analytic approaches (e.g., computational modeling, machine learning, and dynamic structural equation modeling) that can model intensive longitudinal data and more closely reflect the complex and dynamic system of antecedents and consequences involved in contingency learning models of aggression. Together, these expansions have the potential to increase the precision of current intervention efforts designed to reduce aggression by creating a framework for systematically mapping within-individual and within-dyad processes that elicit and maintain aggressive behavior over time.

## Data Availability

No data was used for the research described in the article.

## Footnotes

1 The primary outcome of interest in this study was callous-unemotional traits and aggression was included only as a covariate. Thus, reported findings were correlational and presented as a part of descriptive statistics.

2 While an in-depth overview of differential equations is beyond the scope of the present review, we refer readers to recently published tutorials on differential equations in psychological research (Mongin et al., 2022).

3 AUC stand for area under the curve and is commonly used in machine learning analyses to quantify predictive accuracy. AUC values can range from 0 to 1, with 1 being perfect predictive accuracy. A value of.50 indicates that the predictive accuracy is no better than randomly guessing.

4 The implementation of DSEM in the popular software package MPlus (Muthén & Muthén, 2017) comes with many built-in features that greatly enhance the capabilities of DSEM. Though we do not go into detail here, these built-in default options in MPlus include Bayesian estimation (Asparouhov & Muthén, 2010), the use of latent-mean centering (Asparouhov & Muthén, 2019), and the ability to deal with unequally spaced time intervals between repeated observations (Asparouhov et al., 2018).

5 It is worth noting that similar approaches can been used with machine learning models. Researchers have demonstrated that even when overall out-of-sample prediction is satisfactory (*r* = *0*.59 between predicted depressed mood and reported depressed mood), some individuals demonstrate much more predictable patterns of mood and behavior compared to others (Jacobson & Chung, 2020).
